# Supplementary material for: Current global practice and implications for future research on disseminating health research results to study participants: A systematic review
Source: PLoS Med. 2025 Aug 14;22(8):e1004569. doi: 10.1371/journal.pmed.1004569 (PMC12352677; doi:10.1371/journal.pmed.1004569)
Supplement: S1 Table — (DOCX) [file pmed.1004569.s001.docx]

**S1 Table: Search strategy**

**Key words and search string**

The search strategy included the main keywords ‘results dissemination’, ‘research findings’, ‘study participants’ and combined with “OR” and “AND” operators using different keyword variations. The final keyword chain used was: “(result* adj2 (disseminat* or disclos* or feedback or return* or communicat* or inform* or provid* or shar* or reeiv* or send*)) AND (research or trial or study) adj1 (result* or finding*) AND ((trial or study or research) adj2 (participant* or patient* or subject* or volunteer*) OR research community).

The keywords were adjusted depending on the database searched.

1. **Search strategy in Medline (OVID)**

| **#** | **Searches** | **Results** | **Type** |
| --- | --- | --- | --- |
| 1 | (result*adj2 (disseminat* or disclos* or feedback or return* or communicat* or inform* or provid* or shar* or receiv* or send*)).mp. | 238002 | Advanced |
| 2 | ((research or trial or study) adj1 (result* or finding*)).mp. | 117267 | Advanced |
| 3 | ((trial or study or research) adj2 (participant* or patient* or subject* or volunteer*)).mp. | 443886 | Advanced |
| 4 | Research community.mp. | 10214 | Advanced |
| 5 | 3 or 4 | 453817 | Advanced |
| 6 | 1 and 2 and 5 | 506 | Advanced |
| 7 | Limit 6 to English language | 499 | Advanced |
| 8 | Limit 7 to yr=”2008-current” | 447 | Advanced |

1. **Search strategy in Embase**

| **#** | **Searches** | **Results** | **Type** |
| --- | --- | --- | --- |
| **1** | (result*adj2 (disseminat* or disclos* or feedback or return* or communicat* or inform* or provid* or shar* or receiv* or send*)).mp. | 308136 | Advanced |
|  | ((research or trial or study) adj1 (result* or finding*)).mp. | 227753 | Advanced |
|  | ((trial or study or research) adj2 (participant* or patient* or subject* or volunteer*)).mp. | 832173 | Advanced |
|  | Research community.mp. | 11905 | Advanced |
|  | 3 or 4 | 843605 | Advanced |
|  | 1 and 2 and 5 | 841 | Advanced |
|  | Limit 6 to English language | 835 | Advanced |
|  | Limit 7 to yr=”2008-current” | 770 | Advanced |

1. **search strategy in CINAHL**

| **#** | **Searches** | **Search options** | **Type** |
| --- | --- | --- | --- |
| S1 | (result*N2 (disseminat* or disclos* or feedback or return* or communicat* or inform* or provid* or shar* or receiv* or send*)) | Search modes: Boolean/Phrase | 54094 |
| S2 | ((research or trial or study) N1 (result* or finding*)) | Search modes: Boolean/Phrase | 115447 |
| S3 | ((trial or study or research) N2 (participant* or patient* or subject* or volunteer*)) | Search modes: Boolean/Phrase | 326909 |
| S4 | Research community | Search modes: Boolean/Phrase | 14592 |
| S5 | S3 or S4 | Search modes: Boolean/Phrase | 339684 |
| S6 | S1 and S2 and S5 | Search modes: Boolean/Phrase | 812 |
| S7 | S1 and S2 and S5 | Narrow by Language: English  Search modes: Boolean/Phrase | 793 |
| S8 | S1 and S2 and S5 | Limiters: Publication date 20080101-20241231  Narrow by Language: English  Search modes: Boolean/Phrase | 728 |
